# Evaluation of the TiaoShenZhiAi Regimen for Patients With Ovarian Cancer Experiencing a Psychoneurological Symptom Cluster: Protocol for a Multicenter, Double-Blind, Randomized Controlled Trial

**DOI:** 10.2196/88625

**Published:** 2026-06-12

**Authors:** Ze Liu, Jialiang Yao, Xinyi Lu, Yun Yang, Bin Luo, Yanhong Wang, Xi Cheng, Wei Jiang, Guowang Yang, Jing Xiao, Jiuda Zhao, Jianhui Tian

**Affiliations:** 1 Clinical Cancer Center, Shanghai Municipal Hospital of Traditional Chinese Medicine, Shanghai University of Traditional Chinese Medicine, No. 274, Zhijiang Middle Road, Jing'an District, Shanghai, 200071, China, +86 13761351319; 2 Oncology Institute of Traditional Chinese Medicine, Shanghai Municipal Hospital of Traditional Chinese Medicine, Shanghai, China; 3 Gynecologic Oncology, Fudan University Shanghai Cancer Center, Shanghai, Shanghai, China; 4 Department of Gynecologic Oncology, Obstetrics and Gynecology Hospital of Fudan University, Shanghai, Shanghai, China; 5 Department of Oncology, Beijing Hospital of Traditional Chinese Medicine, Beijing, Beijing, China; 6 Department of Gynecology, Guangdong Provincial Hospital of Traditional Chinese Medicine, Guangzhou, Guangdong, China; 7 Breast Disease Diagnosis and Treatment Center, Qinghai University Affiliated Hospital, Xi'ning, Qinghai, China

**Keywords:** ovarian cancer, psychoneurological symptom cluster, TiaoShenZhiAi, TSZA regimen, randomized controlled trial, clinical trial protocol, traditional Chinese medicine

## Abstract

**Background:**

Psychoneurological symptom clusters (PNSCs) are common in patients with ovarian cancer and are associated with reduced quality of life, treatment interruption, and poor prognosis. However, effective interventions for PNSCs remain limited. Traditional Chinese medicine may provide comprehensive benefits for symptom management.

**Objective:**

This study aims to evaluate the efficacy and safety of the TiaoShenZhiAi (TSZA) regimen in alleviating PNSCs in patients with ovarian cancer and to assess its effects on quality of life and survival outcomes.

**Methods:**

A total of 316 patients with ovarian cancer aged 18 to 70 years with PNSCs will be included and randomly divided into 2 parallel groups. Both groups will receive standard treatment for ovarian cancer as the basic treatment. The intervention group will receive the TSZA regimen, that is, Compound Ciwujia Granules (containing *Acanthopanax senticosus* and *Schisandra chinensis*) combined with psychological intervention. The control group will receive a low-dose active control (simulated Compound Ciwujia Granules) combined with psychological intervention. The primary outcome is the remission rate of PNSCs at 3 months. The secondary outcome measures include the Pittsburgh Sleep Quality Index, the Patient Health Questionnaire-9, the Generalized Anxiety Disorder-7 scale, the revised Piper Fatigue Scale, the European Organization for Research and Treatment of Cancer Quality of Life Questionnaire - Core 30 Quality of Life Scale, the traditional Chinese medicine syndrome scale, sleep quality, sleep diary, and the 1-year survival analysis. In addition, this study also includes a series of exploratory indicators (including functional magnetic resonance imaging, biomarkers of peripheral blood and tumor tissue, proportion of immune cells, cytokine levels, hypothalamic-pituitary-adrenal axis function, and immune gene expression analysis) and safety indicators (including vital signs, liver and kidney function, and electrocardiogram). The study outcomes will be evaluated based on different indicators during the treatment period (baseline and the 1st, 2nd, and 3rd mo of enrollment) and the follow-up period (the 6th, 9th, and 12th mo of enrollment). Data analysis will be conducted using R (version 4.5.3) software. A one-sided *P* value of <.03 will be considered statistically significant.

**Results:**

This study is designed to enroll a total of 316 participants. Participant enrollment is set to commence in October 2025, with no recruitment having occurred as of April 2026. The recruitment period will extend until September 2028 or until the target enrollment is met. Data analysis is scheduled for November 2028, with submission of the trial results to a peer-reviewed journal anticipated by May 2029.

**Conclusions:**

This study will evaluate the efficacy of the TSZA regimen in managing PNSCs in patients with ovarian cancer and generate clinical evidence for a new therapeutic option that improves quality of life and alleviates the symptom burden.

## Introduction

### Background

Malignant tumors represent a major global public health challenge. According to the latest World Health Organization (WHO) statistics, cancer is the second leading cause of death worldwide. The global burden of cancer continues to rise [1], with gynecological malignancies—such as ovarian cancer and cervical cancer—constituting a substantial proportion of cancer-related incidence and mortality among women. Although current comprehensive treatment strategies have significantly improved patient survival, multiple symptoms resulting from both the disease and antitumor therapies continue to severely affect patients’ quality of life, treatment compliance, and long-term prognosis [2,3].

Since the initial description of symptom co-occurrence in patients with cancer [4], the concept has evolved into the “symptom cluster,” defined as a stable group of 2 or more interrelated symptoms that often share underlying mechanisms and result in clinical outcomes more severe than the sum of individual symptoms [5-9]. Despite the lack of a singular consensus on its definition, it is widely recognized that symptom clusters are complex and heterogeneous. Their presence is strongly associated with diminished quality of life, increased risk of treatment interruption, and poorer prognosis, necessitating a shift from single-symptom management to a more holistic, cluster-based intervention approach.

Currently, intervention strategies for symptom clusters primarily fall into 2 categories: pharmacological and nonpharmacological treatments. Pharmacological management follows the principle of symptomatic support, wherein specific medications are selected based on the patient’s predominant symptoms—for instance, analgesics for pain relief or anxiolytic and antidepressant agents for alleviating anxiety and depression. Nonpharmacological interventions include a variety of approaches such as structured exercise, mindfulness meditation, professional psychological support, and music therapy, among others [10-12]. Although these interventions have become integral to comprehensive symptom management, they tend to emphasize general applicability and have yet to evolve into systematic protocols tailored to specific cancer types. This lack of personalized and structured approaches may limit further improvement in therapeutic outcomes.

Surgery remains the cornerstone of treatment for ovarian cancer. However, bilateral oophorectomy induces a precipitous decline in estrogen levels, leading to a constellation of sequelae, such as premature menopausal symptoms, sexual dysfunction, and altered body image [13,14]. The loss of reproductive organs can profoundly affect a woman’s self-identity, body perception, and gender identity [15], often resulting in significant psychological distress [16]. This internal conflict is frequently exacerbated by a discrepancy between societal stereotypes of femininity and the patients’ lived experiences, which can contribute to social withdrawal and strained family relationships [17].

Furthermore, the clinical prognosis adds another layer of complexity. Approximately 80% of patients with ovarian cancer are diagnosed at an advanced stage, with approximately 75% experiencing disease recurrence within 2 years. The 5-year overall survival (OS) rate for advanced-stage disease remains dismally low, ranging from 10% to 40% [18]. This stark clinical reality imposes a substantial psychological burden on patients. Compounding this are therapeutic challenges—including platinum resistance, limited targetable mutations in homologous recombination repair pathways, and an immunologically inert tumor microenvironment—which further intensify psychological distress in patients from a prognostic standpoint [19].

In summary, the interplay of surgical, psychological, social, and prognostic factors creates a complex web of stressors that collectively contribute to the high prevalence of psychoneurological symptom clusters (PNSCs) in patients with ovarian cancer.

Traditional Chinese medicine (TCM) emphasizes holistic regulation, with accumulating evidence demonstrating its efficacy in managing cancer-related symptom clusters. For instance, acupuncture significantly alleviates chemotherapy-induced nausea and vomiting [20] while also improving clusters comprising pain, fatigue, and sleep disturbances [21]. Furthermore, TCM interventions effectively relieve menopausal-like symptoms—such as hot flashes and insomnia—arising from treatment-induced endocrine deficiencies [22]. Mind-body exercises such as Tai Chi have even exhibited therapeutic effects comparable to cognitive behavioral therapy (CBT) for insomnia [23].

Building on these foundations, together with insights from our team’s clinical experience and preliminary small-scale studies, we have developed an integrative interventional protocol named the “TiaoShenZhiAi” (TSZA) regimen. Rooted in the TCM philosophy of “Regulating the Spirit to Treat Cancer,” the TSZA regimen is designed as an interdisciplinary oncological approach targeting psychoneurological symptoms associated with cancer, with the aim of improving patient survival and quality of life. In this study, the TSZA regimen specifically refers to a combination of standard-dose Compound Ciwujia Granules and a structured psychological intervention.

We have initiated this multicenter randomized controlled trial to rigorously evaluate the efficacy of the TSZA regimen in patients with ovarian cancer and PNSCs. Beyond clinical symptom remission, this study aims to enhance treatment adherence and potentially extend survival. Crucially, by integrating multimodal neuroimaging (functional magnetic resonance imaging [fMRI]) and targeted proteomics, we seek to elucidate the underlying biological signatures of PNSCs and the restorative mechanisms of the TSZA regimen, thereby advancing precision intervention strategies in integrative oncology.

### Objectives

The primary objective of this study is to evaluate the efficacy and safety of the TSZA regimen in alleviating PNSCs among patients with ovarian cancer. The secondary objectives include assessing its impact on patients’ quality of life and survival outcomes. Furthermore, the study aims to explore the characteristics of the patient population that derives benefit from the TSZA regimen for these symptoms.

## Methods

### Trial Design

**Figure 1.**
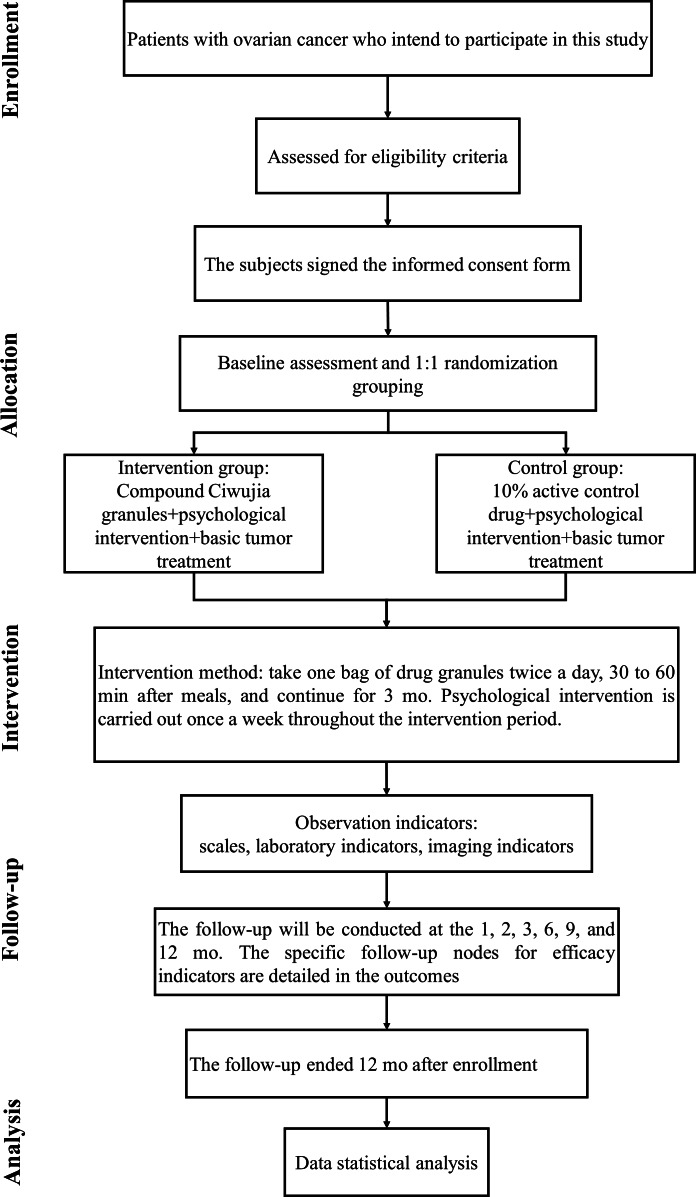
Study flowchart.

This study uses a randomized, double-blind, active-controlled (low-dose control), prospective, multicenter design. Eligible participants will be randomized in a 1:1 ratio to either the intervention group or the control group. Prior to enrollment, baseline data—including medical history, physical examination findings, relevant laboratory results, and patient-reported outcomes—will be collected for screening and eligibility confirmation. All enrolled participants will be managed under a unified protocol. The combined drug and psychological intervention will be administered over a 3-month period. On completion of the intervention, participants will undergo 3 subsequent follow-up visits, each spaced 3 months apart, resulting in a total follow-up duration of 12 months. Figure 1 presents the participant flow through the trial.

### Participant Selection

Participants will be recruited from 6 hospitals in mainland China. Eligible individuals must be patients diagnosed with ovarian cancer accompanied by PNSCs. The diagnosis of ovarian cancer will be based on the 2020 WHO Classification of Female Genital Tumours (5th edition) and will be confirmed by cytological or histopathological examination. Disease staging will follow the 2017 International Federation of Gynecology and Obstetrics staging system (8th edition). The presence of a PNSC will be assessed using the MD Anderson Symptom Inventory for Ovarian Cancer (MDASI-OC) scale, a validated instrument for evaluating symptoms in patients with ovarian cancer. According to the standard definition of symptom clusters, 4 related symptoms—fatigue, sleep disturbance, distress, and sadness—will be evaluated. An average score of ≥4 across these 4 symptoms will be required to define the presence of the PNSCs. Recruitment will be conducted through multiple channels, including physician referrals, patient referrals, and posted advertisements in participating hospitals. Table 1 presents the specific information of participating units.

**Table 1.** The 6 participating centers.

Center	Department	Investigator
Shanghai Hospital of Traditional Chinese Medicine	Clinical Research Center for Oncology	JT
Fudan University Shanghai Cancer Center	Gynecologic Oncology	XC
Obstetrics and Gynecology Hospital of Fudan University	Gynecologic Oncology	WJ
Beijing University of Chinese Medicine Hospital Affiliated to Capital Medical University	Department of Oncology	GY
Guangdong Provincial Hospital of Chinese Medicine	Gynecology	JX
Qinghai University Affiliated Hospital	Breast Disease Diagnosis and Treatment Center	JZ

### Eligibility Criteria

#### Inclusion Criteria

The inclusion criteria are as follows:

1. Histologically or cytologically confirmed primary epithelial ovarian cancer.
2. Meeting the diagnostic criteria for chronic insomnia defined by the Sleep Disorders Group of the Neurology Branch of the Chinese Medical Association, including a Pittsburgh Sleep Quality Index total score >8, a Piper Fatigue Scale total score >4, and a Patient Health Questionnaire-9 total score >5.
3. Moderate-to-severe PNSC severity. PNSC will be assessed using the MDASI-OC. Moderate-to-severe PNSC is defined as a mean score of ≥4 across the 4 items: sleep disturbance, fatigue, distress, and sadness.
4. Eastern Cooperative Oncology Group Performance Status score of 0 to 2.
5. Age 18 to 70 years.
6. Meeting the TCM diagnostic criteria for spleen-kidney yang deficiency syndrome.
7. Expected survival >1 year.
8. Signed informed consent form with voluntary acceptance of the treatment protocol and ability to independently complete sleep diaries.

#### Exclusion Criteria

The exclusion criteria are as follows:

1. Patients scheduled to undergo radiotherapy within the next 4 treatment cycles
2. Comorbid severe primary diseases of the heart, brain, liver, kidney, or hematopoietic system, including hepatic dysfunction (aspartate aminotransferase or alanine aminotransferase >1.5 times the upper limit of normal) or renal impairment (serum creatinine >1.2 times the upper limit of normal)
3. Pregnant or lactating women, individuals with psychiatric disorders (eg, schizophrenia, bipolar disorder, mania, depressive disorder, anxiety disorders, and phobias), intellectual or language impairments, or other mental health conditions
4. Scores ≥15 on the Patient Health Questionnaire for depression or ≥15 on the Generalized Anxiety Disorder-7 at screening
5. Preexisting chronic insomnia or depression diagnosed prior to ovarian cancer
6. Comorbid autoimmune diseases, hematologic disorders, or long-term use of corticosteroids or immunosuppressants
7. History of other primary malignancies
8. Participation in other clinical trials within 3 months
9. HIV-positive status, congenital or acquired immunodeficiency disorders, or history of organ transplantation (including autologous bone marrow or peripheral stem cell transplantation)
10. Legally incapacitated individuals or cases with medical or ethical contraindications to study continuation
11. Active hepatitis B, active tuberculosis, or evidence of severe or uncontrolled systemic inflammatory conditions (eg, unstable respiratory, cardiovascular, hepatic, or renal diseases)
12. Patients with diabetes

#### Dropout Criteria

The dropout criteria are as follows:

1. Occurrence of complications, comorbidities, or serious adverse events (SAEs) making continued participation in the trial inadvisable.
2. Investigator determination that discontinuation or suspension of the clinical trial is warranted for safety reasons.
3. Withdrawal from the study by the subject for any reason.
4. Participants retain the right to withdraw from the trial at any time. Investigators should document the reason for withdrawal to the extent possible.

### Sample Size

A superiority design is used for this study. Given the lack of a universally established remission standard for the PNSCs derived from the MDASI-OC scale, parameters are determined based on internal pilot data and expert consensus. The remission rate for the control group at 3 months is estimated at 10%. We posited an absolute increase of 30% in the intervention group, corresponding to an expected remission rate of 40%. To ensure clinical significance, a superiority margin (*M*) of 15% is prespecified and incorporated into the sample size formula. Assuming a one-sided significance level (α) of .025 and a statistical power (1-β) of .80, calculations performed via PASS software (version 15.0; Superiority by a Margin Tests for the Difference Between Two Proportions) yielded a required sample size of 116 participants per group. Accounting for an anticipated 20% dropout rate, a minimum of 145 participants per group is required. To further enhance the robustness of the findings and to accommodate potential variability in dropout rates or covariate adjustments, the final total sample size is determined to be 316 participants (158 per group). This ensures that the actual statistical power of the final analysis set remains consistently above 80%.

### Randomization

After registration and completion of the baseline assessment, eligible patients who provide written informed consent will be randomly assigned in a 1:1 ratio to either the intervention group or the control group. Randomization will be performed using a centralized system using center-stratified block randomization. An independent statistician, who is not involved in the trial implementation, will generate the allocation sequence. The randomization results will be encrypted and transmitted to an independent third party responsible for the allocation system, where they will be kept confidential until trial completion. The personnel responsible for participant recruitment and intervention delivery will remain blinded to the group assignments.

### Blinding

This study adopts a double-blind design. Drug blinding and distribution packaging are jointly carried out by statisticians who are not directly involved in this clinical trial and the pharmaceutical factory responsible for drug production. The trial drug and the control drug are respectively assigned unique drug packaging numbers and drug blinding codes, which are filled in (or pasted) on the drug labels. The control drug contains 10% of the components of the investigational drug. This active-controlled design provides a more rigorous comparison and addresses the practical challenges of producing an indistinguishable placebo in TCM research.

An independent blind background file will be established for each random number. The content includes key information such as the group to which the subject with the number is assigned (such as group A or group B) and the medication requirements (investigational drug or control drug) in case of emergency unblinding. The third party responsible for the random system is responsible for confidential storage. It is only opened by authorized personnel in accordance with the procedures in emergency situations, such as SAE, where unblinding is necessary.

A 2-step unblinding method will be adopted. After all research data are entered, verified, and have undergone blinded review, the database will be locked. The first unblinding will be performed by the blinding code custodian to identify group A and group B. This information will then be handed over to the biostatistician for statistical analysis. After the statistical analysis is completed, the second unblinding will be conducted to clarify the intervention and control groups.

### Interventions

In this trial, both the intervention and control groups will receive standard-of-care treatment for ovarian cancer as the foundational therapy, in accordance with the 2024 NCCN International Guidelines. This may include platinum-based chemotherapy and/or targeted, antiangiogenic, or hormonal agents, as clinically indicated.

Both the intervention and control groups will receive the same psychological intervention. The intervention is based on CBT and mindfulness theory and is adaptively designed to address the characteristics of the PNSC in patients with ovarian cancer. It comprises a total of 12 sessions. The intervention is delivered in an online prerecorded format, once weekly, with each session lasting approximately 30 to 45 minutes. The materials are distributed to participants on schedule by the investigators according to a treatment manual. The session content includes cognitive restructuring of illness perceptions, pain tolerance, understanding and expressing interpersonal needs, family and social support, comorbidity of depression and sleep disturbances, sleep hygiene education, the theoretical basis of insomnia, stimulus control, cognitive restructuring for insomnia, emphasis on long-term coexistence, finding meaning within limitations, and relapse prevention. All sessions will be recorded by qualified mental health professionals from the Shanghai Mental Health Center, China. Prior to release, each session script is independently reviewed by another psychotherapist or the principal investigator to ensure objectivity, avoid leading statements or overpromising, and comply with ethical requirements for psychooncological interventions. To ensure consistency of intervention delivery across centers, standardized prerecorded materials are used at all sites. Investigators at each center receive unified training prior to study initiation, and intervention adherence is centrally monitored and recorded by the study team.

In addition, participants in the intervention group will receive the TSZA regimen, which consists of Compound Ciwujia Granules (containing *Acanthopanax senticosus* and *Schisandra chinensis*) combined with psychological intervention. The granules will be administered twice daily, one sachet each time, 30 to 60 minutes after meals, for a continuous period of 3 months.

The control group will receive matching low-dose active control granules (containing 10% of the active ingredients found in Compound Ciwujia Granules), which are identical in appearance, odor, and taste to the investigational drug, following the same dosing schedule.

During the study period, the use of any medications not specified in the study protocol is prohibited. In principle, participants should not take other Chinese herbal medicines, Chinese patent drugs with sedative or sleep-promoting claims, biologics, or any other agents that may potentially affect sleep, fatigue, or mental health. Any use must be reported.

### Follow-Up

During the 3-month treatment period, follow-up visits will be scheduled once per month. After the completion of treatment, follow-up assessments will be performed every 3 months. The total follow-up duration will be 1 year. Data collection time points are presented in Table 2.

**Table 2.** Study timeline.

Study stage	Screening and baseline period	Treatment period			Follow-up period		
Visit (times)	0	1	2	3	4	5	6
Time point (day)	−14 to 0	30	60	90	180	270	360
Time window (day)	+3 or −3	+3 or −3	+3 or −3	+3 or −3	+7 or −7	+7 or −7	+7 or −7
Collection of basic medical history							
Informed consent	✓						
Emission standards	✓						
Demographic data	✓						
Diagnosis and treatment history	✓						
Assessments of validity							
MDASI-OC^a^ scale	✓	✓	✓	✓	✓	✓	✓
PSQI^b^ sleep scale	✓	✓	✓	✓	✓	✓	✓
PFS-R^c^ scale	✓	✓	✓	✓	✓	✓	✓
PHQ-9^d^ depression scale	✓	✓	✓	✓	✓	✓	✓
GAD-7^e^ anxiety scale	✓	✓	✓	✓	✓	✓	✓
EORTC QOL-C30^f^ scale	✓	✓	✓	✓	✓	✓	✓
TCM^g^ syndrome scale	✓	✓	✓	✓	✓	✓	✓
One-year OS^h^ rate and PFS^i^ rate							✓
Sleep quality	✓	✓	✓	✓			
Sleep diary	✓	✓	✓	✓			
fMRI^j^	✓			✓			
Biological targets in peripheral blood	✓			✓			
Biological targets in tumor tissue	✓						
Tumor markers	✓			✓			
Proportions of immune cells	✓			✓			
Cytokine levels	✓			✓			
HPA^k^ axis function	✓			✓			
Analysis of immune gene expression	✓			✓			
Vital signs	✓	✓	✓	✓	✓	✓	✓
Laboratory Test Indicators and ECG^l^	✓			✓			
AE^m^ and SAE^n^	✓	✓	✓	✓	✓	✓	✓

a MDASI-OC: MD Anderson Symptom Inventory for Ovarian Cancer.

b PSQI: Pittsburgh Sleep Quality Index.

c PFS-R: Piper Fatigue Scale-Revised.

d PHQ-9: Patient Health Questionnaire-9.

e GAD-7: Generalized Anxiety Disorder-7.

f EORTC QOL-C30: European Organisation for Research and Treatment of Cancer Quality of Life Questionnaire - Core.

g TCM: traditional Chinese medicine.

h OS: overall survival.

i PFS: progression-free survival.

j fMRI: functional magnetic resonance imaging.

k HPA: hypothalamic-pituitary-adrenal.

l ECG: electrocardiography.

m AE: adverse event.

n SAE: serious adverse event.

### Outcomes

#### Primary Outcome

The PNSC score is constructed based on the 4 most relevant symptoms (fatigue, sleep disturbance, distress, and sadness) from the MDASI-OC scale, in accordance with its usage guidelines [24]. Each symptom is rated on an 11-point scale, ranging from 0 (“no symptom”) to 10 (“the most severe imaginable”). The primary efficacy end point of this study is the difference in the PNSC remission rates between the intervention and control groups at 3 months after enrollment. Given that there is no universally accepted evaluation standard for the remission of this specific cluster, “remission” is defined in this protocol through expert consensus. A participant is classified as in “remission” if they meet at least one of the following PNSC-specific criteria: a ≥50% reduction in the total PNSC score relative to the baseline or an absolute PNSC score falling below the threshold of 4 after the intervention. While the primary comparison between the 2 groups will focus on the 3-month mark, longitudinal assessments will be conducted at baseline and at 1, 2, 3, 6, 9, and 12 months after enrollment to monitor symptom dynamics.

#### Secondary Outcomes

##### Pittsburgh Sleep Quality Index

This scale is a widely used instrument comprising 19 self-rated items (which are scored) and 5 nonscored, other-rated items. The 19 scorable items are grouped into 7 components: subjective sleep quality, sleep latency, sleep duration, habitual sleep efficiency, sleep disturbances, use of sleeping medication, and daytime dysfunction. The global Pittsburgh Sleep Quality Index score ranges from 0 to 21, with a higher total score indicating poorer sleep quality [25]. Assessments will be conducted at baseline and at 1, 2, 3, 6, 9, and 12 months after enrollment.

##### Patient Health Questionnaire-9

This scale comprises 9 items that evaluate low mood, loss of interest, sleep disturbance, fatigue, appetite changes, feelings of worthlessness or guilt, concentration difficulties, psychomotor agitation or retardation, and suicidal ideation. Patients will rate the frequency of each symptom over the past 2 weeks on a scale from 0 (“not at all”) to 3 (“nearly every day”). The total score ranges from 0 to 27, with higher scores indicating more severe depressive symptoms [26]. Assessments will be conducted at baseline and at 1, 2, 3, 6, 9, and 12 months after enrollment.

##### Generalized Anxiety Disorder-7 Scale

This scale will be used to screen for and assess the severity of generalized anxiety disorder. It consists of 7 items, each rated on a 4-point scale from 0 (“not at all”) to 3 (“nearly every day”). The total score ranges from 0 to 21, with higher scores indicating more severe anxiety symptoms [27]. Assessments will be conducted at baseline and at 1, 2, 3, 6, 9, and 12 months after enrollment.

##### Revised Piper Fatigue Scale-Chinese Version

This scale is used to assess fatigue. It is a 22-item instrument derived from the original Piper Fatigue Scale, measuring 4 dimensions: behavioral, affective, sensory, and cognitive. Each item is scored on a scale from 0 to 10. The total fatigue score ranges from 0 to 220, and a mean score is calculated by dividing the total score by 22. Higher scores indicate more severe fatigue [28]. Assessments will be conducted at baseline and at 1, 2, 3, 6, 9, and 12 months after enrollment.

##### The European Organisation for Research and Treatment of Cancer Quality of Life Questionnaire - Core Scale

This core instrument for patients with cancer comprises 30 items. Most items are scored on a 4-point scale (1=not at all to 4=very much). Items 29 and 30, which assess overall health and quality of life, use a 7-point scale (1 to 7). The items form 15 scales: 5 functional scales (physical, role, cognitive, emotional, and social), 3 symptom scales (fatigue, pain, and nausea or vomiting), a global health status or quality of life scale, and 6 single items. All scale scores will be linearly transformed to a 0- to 100-point scale. For the functional and global health status scales, a higher score represents a better level of functioning or quality of life. For the symptom scales and single items, a higher score indicates a greater severity of symptoms or problems [29]. Assessments will be conducted at baseline and at 1, 2, 3, 6, 9, and 12 months after enrollment.

##### The TCM Syndrome Pattern Assessment Scale

It is used to collect participants’ relevant symptoms in accordance with the theoretical system of TCM syndrome differentiation. Assessments will be conducted at baseline and at 1, 2, 3, 6, 9, and 12 months after enrollment.

##### Sleep Quality

Portable wrist actigraphy will be used to record patients’ sleep duration. Assessments will be conducted at baseline and at 1, 2, and 3 months after enrollment.

##### Sleep Diary

Sleep will be subjectively monitored using the Sleep Diary Consensus Core Edition [30], which includes bedtime, sleep onset time, number and duration of nocturnal awakenings, and final wake-up time. Assessments will be conducted at baseline and at 1, 2, and 3 months after enrollment.

##### Survival Period

The 1-year OS and progression-free survival (PFS) rates will be determined based on regular imaging assessments conducted during the 1-year follow-up period after randomization.

### Exploratory Outcomes

#### fMRI Usage

fMRI is an important tool for studying psychoneural mechanisms by detecting blood oxygen level–dependent signals in brain activity and is also an important means for evaluating therapeutic effects [31]. The fMRI will be used to identify the brain regions associated with the PNSCs in ovarian cancer and to assess the changes following treatment. fMRI is planned for 30 randomly selected patients per group, totaling 60 participants. Assessments will be conducted at baseline and 3 months after enrollment.

#### Biological Targets in Peripheral Blood

Differential protein expression will be measured using targeted proteomics in patients with cancer and PNSCs before and after treatment, with the goal of exploring potential actionable therapeutic targets. The study will randomly select 50 cases from each group, totaling 100 patients for examination. Assessments will be conducted at baseline and at 3 months after enrollment.

#### Biological Targets in Tumor Tissue

Some proteins are involved in both the biological processes of tumors and the generation of psychoneurological symptoms [32]. Targeted proteomics will be performed exclusively on tumor tissues obtained before treatment to detect differentially expressed proteins between patients with cancer and PNSCs and cancer-free controls. Additionally, common biological targets in peripheral blood and tumor tissue samples will be explored. The study will randomly select 50 cases from each group, totaling 100 patients for examination. Tumor tissue will be collected at baseline.

#### Tumor Markers

CA125 and HE4—which are routinely used in combination for monitoring ovarian cancer—will serve as the primary monitoring indicators [33]. Additional data on related markers (including CA19-9 and CEA) will be concurrently collected to enable a comprehensive analysis. Assessments will be conducted at baseline and 3 months after enrollment.

#### Proportions of Immune Cells

The proportions of immune cells, such as T cells, B cells, and natural killer cells, will be measured. The study will randomly select 50 cases from each group, totaling 100 patients for examination. Assessments will be conducted at baseline and at 3 months after enrollment.

#### Cytokine Levels

The expression levels of cytokines, such as interleukin-6, interleukin-10, tumor necrosis factor-α, and interferon-γ, will be measured. The study will randomly select 50 cases from each group, totaling 100 patients for examination. Assessments will be conducted at baseline and at 3 months after enrollment.

#### Functional Assessment of the Hypothalamic-Pituitary-Adrenal Axis

Hypothalamic-pituitary-adrenal (HPA) axis function will be assessed by measuring plasma adrenocorticotropic hormone, urine free cortisol, and plasma cortisol levels. Adrenocorticotropic hormone is a pivotal mediator within the axis, while cortisol serves as its primary effector hormone; elevated cortisol levels typically indicate a physiological stress state [34]. Fifty cases will be randomly selected from each group, totaling 100 patients for testing. Assessments will be conducted at baseline and at 3 months after enrollment.

#### Analysis of Immune Gene Expression

RNA sequencing analysis will be performed on patient-derived biological samples to investigate expression changes in immune-related genes, with a specific focus on their roles in immune regulation. This study will randomly select 50 cases from each group, totaling 100 patients for research. Assessments will be conducted at baseline and at 3 months after enrollment.

### Safety Outcomes

Vital signs (respiration, body temperature, heart rate, and blood pressure) will be monitored at baseline and at 1, 2, 3, 6, 9, and 12 months after enrollment. Laboratory test indicators—including complete blood count (white blood cells, hemoglobin, and platelets), urinalysis (urine protein and urine white blood cells), liver and kidney function (alanine aminotransferase, aspartate aminotransferase, and serum creatinine), and electrocardiogram (ECG [electrocardiography] findings and QT interval)—will be assessed at baseline and at 3 months. All findings will be categorized based on clinical significance as normal, abnormal but not clinically significant, or abnormal and clinically significant.

### Adverse Events

Adverse events (AEs) are defined as any undesirable or negative experience or outcome that does not have a causal relationship with the treatment. Although the treatment regimens used in clinical research have undergone initial safety evaluations, individual differences may lead to unexpected adverse reactions in some participants. All AEs will be recorded, and appropriate management will be immediately provided. Furthermore, participants are guaranteed by state health insurance and are permitted to withdraw from the study at any stage without any consequences.

### Qualification of the Investigators

Researchers are required to have relevant qualifications or experience in conducting medical clinical research. Before the start of the study, the researchers will receive training to be familiar with the research plan, correctly master the inclusion and exclusion standards and scale evaluation methods, and fill in the Electronic Data Capture (EDC) in a standardized manner. In addition, psychological intervention training will be provided by mental health experts.

### Data Collection and Management

#### Data Entry and Modification

Data collection, entry, and modification will be conducted using the EDC and electronic Patient-Reported Outcome systems. The study will provide all participants with protocol-required laboratory and imaging tests at no cost, along with a fixed travel allowance for follow-up visits, to enhance patient adherence to follow-up visits. Participants who discontinue the study intervention or deviate from the protocol will be withdrawn from the study. Only the data from the last follow-up visit prior to withdrawal will be recorded. Two data managers will be responsible for data entry and verification. For questions in the EDC, the data manager will revise and confirm the data according to the original data of patients and the answers of researchers to ensure the overall data quality. Upon completion of data entry, consistency checks will be performed, and the database will be locked.

#### Missing Value Handling

Missing data will be handled according to the type and role of each variable. For missing diagnosis dates, original pathology records will be retrieved whenever possible. If the pathology report date is unavailable, the date of surgery will be used for imputation. If neither source can be obtained, the date of the patient’s first visit for ovarian cancer will be used, with the month imputed as July of that year and the day as the first day of the month. For baseline demographic and clinical characteristics, no imputation will be performed, and available data will be summarized descriptively. For safety outcomes, only observed AEs will be included in the analysis without imputation. For primary and secondary efficacy end points, missing follow-up data will be handled using multiple imputation under the missing at random assumption. The imputation model will include treatment group, baseline outcome values, and key prognostic covariates. Complete case analysis will be performed as a sensitivity analysis to assess the robustness of the primary findings.

#### Data Monitoring

The data monitoring committee for this experiment will be made up of 4 experts who are not involved in the research team. The committee will be in charge of external oversight, monitoring the study’s viability, data integrity, and safety. A risk-based monitoring approach will be adopted, primarily through random audits. Data with poor compliance or containing modifications in the EDC system will be subject to targeted review.

AEs must be documented in detail, appropriately managed, and tracked until resolution or stabilization. SAEs and unanticipated problems shall be reported in a timely manner to the Ethics Committee, the competent regulatory authority, the sponsor, and other relevant oversight bodies as required.

Study progress reports shall be submitted on schedule, including explanations for any AEs, subject withdrawals, and protocol deviations. The principal investigator will periodically conduct cumulative reviews of all AEs and, when necessary, convene investigator meetings to evaluate the risk-benefit profile of the study.

#### Data Preservation

All trial data, including documented participant eligibility, original signed informed consent forms, and detailed records of investigational product distribution and retrieval, will be maintained by the research team.

### Data Analysis

#### Final Analysis

Data analyses will be performed using R (version 4.5.3 or later).

This study defines 2 analysis sets: the full analysis set (FAS) and the per-protocol set. The primary efficacy analysis will be conducted based on the FAS, following the intention-to-treat principle, including all eligible participants with available baseline data. The per-protocol set will be used for sensitivity analyses to assess the robustness of the results. Demographic and baseline characteristics will be summarized using the FAS. Safety outcomes will be summarized descriptively.

#### Missing Data Analysis

To maintain the integrity of the intention-to-treat analysis, missing data for primary and secondary efficacy end points arising during follow-up will be handled using multiple imputation under the missing at random assumption. This ensures that all randomized participants are included in the final analysis, minimizing potential bias from attrition.

#### Descriptive Statistics

Continuous variables will be described as mean (SD) or median (IQR), as appropriate. Categorical variables will be presented as frequencies and percentages.

#### Primary Outcome Analysis

The primary end point is the PNSC remission rate at 3 months after enrollment. The chi-square test will be used to analyze whether the difference in remission rates between the two groups is statistically significant. Meanwhile, the difference in remission rates between the experimental group and the control group, along with the 95% CI, will be calculated. In addition, to account for potential between-group heterogeneity, a binary logistic regression model will be performed, with group as the main independent variable and baseline PNSC scores included as covariates. Study center will be included as a fixed effect to account for potential between-center heterogeneity. In addition, longitudinal PNSC scores will be analyzed using the methods described below for repeated continuous outcomes.

#### Secondary Outcome Analysis

Linear mixed effects models will be used to analyze continuous variables with repeated measures (eg, scale scores at multiple time points). The models will include group, time, and their interaction as fixed effects; individual subject IDs and study centers will be incorporated as random effects.

#### Survival Outcomes

One-year OS and PFS will be analyzed using the Kaplan-Meier method with log-rank tests. A Cox proportional hazards model will be used to estimate hazard ratios.

A one-sided *P* value of <.03 will be considered statistically significant.

### Interim Analysis

One interim analysis is planned when approximately 50% of participants have completed the 3-month primary end point assessment. To control the overall one-sided type I error rate at 0.025, a group sequential approach based on the O’Brien-Fleming alpha-spending function will be applied, allocating one-sided nominal significance levels of 0.0026 and 0.0240 to the interim and final analyses, respectively.

#### Sample Size Reestimation

An internal pilot design will be used for sample size reestimation based on the observed effect size at the interim stage. If the observed effect is smaller than initially assumed but still clinically relevant, the sample size will be adjusted using the formula: $N_{R}={\left(E_{0}/E\right)}^{2}\times N_{0}$, where $N_{R}$ is the adjusted sample size (capped at $2N_{0}$). On the basis of interim results, the Independent Data Monitoring Committee will evaluate the conditional power of the trial. If the power is insufficient, the committee will discuss the necessity of sample size adjustment or early termination.

#### Formal Stopping Rules

The trial may be terminated early for overwhelming efficacy if the interim test statistic crosses the prespecified O’Brien-Fleming boundary (corresponding to a one-sided significance level of 0.0026), or for futility based on conditional power assessment, as determined by the Independent Data Monitoring Committee.

### Participants Research Protection

While there is a certain risk of information leakage, we have implemented comprehensive measures to mitigate such risks. EDC system is secured via username and password authentication, with all patient information being encrypted to ensure that only authorized research personnel and regulatory officials can access participant data. Individuals will be identified solely by a unique ID number and initials in all data analyses and publications.

Following the trial, participants who demonstrate a positive response to the investigational product may choose to continue its use at their own discretion. For those with a suboptimal response, other appropriate therapeutic options will be recommended based on their specific clinical presentation.

Additionally, should any participant sustain harm during the trial that is medically determined to be related to the study, the sponsor will provide appropriate compensation.

### Dissemination Plans

The findings of this study may be submitted for publication in peer-reviewed medical journals, conference presentations, broadcast media, and presentations to stakeholders. In all publications, participant confidentiality will be strictly protected in accordance with applicable laws and regulations. No personally identifiable information will be disclosed unless mandated by law.

### Ethical Considerations

This study will be conducted in accordance with the principles of the Declaration of Helsinki and relevant Chinese laws, regulations, and guidelines pertaining to clinical trials. The study protocol has been approved by the Ethics Committee of Shanghai Hospital of Traditional Chinese Medicine (Approval ID: 2024SHL-KY-108‐02). Any protocol amendments must be submitted to the lead Ethics Committee for approval. Once approved, they shall then be submitted to the ethics committees of all participating sites for filing. After having fully understood the trial’s methodology, objectives, potential benefits, and risks, all eligible participants will provide signed informed consent in person, agreeing to the collection of trial-related subject data and biological specimens.

## Results

This study is designed to enroll a total of 316 participants. Participant enrollment is set to commence in October 2025, with no recruitment having occurred as of April 2026. The recruitment period will extend until September 2028 or until the target enrollment is met. Data analysis is scheduled for November 2028, with submission of the trial results to a peer-reviewed journal anticipated by May 2029.

## Discussion

This study is a multicenter, randomized, double-blind clinical trial with a low-dose positive control, and it will enroll 316 patients with ovarian cancer and PNSCs. The aim is to test the effect of the TSZA regimen on the symptom cluster of fatigue, insomnia, anxiety, and depression. The study also follows survival and measures inflammatory factors, immune function, neuroimaging, and multiomics to confirm its clinical value and explore its biological mechanism.

As noted earlier, the high rate of PNSC in patients with ovarian cancer is not due to one factor but results from surgery, chemotherapy, psychological stress, lack of social support, and uncertain prognosis. These factors form a complex stress network and trigger a cascade of symptoms [35]. Studi

es show that chronic stress keeps the HPA axis active and disrupts secretion rhythms [36]. This long-term imbalance is a biological basis of PNSC, and it also reduces natural killer cell activity and increases proinflammatory cytokines, which creates an immune-suppressive environment that supports tumor growth and spread [37]. For example, basic research shows that chronic stress increases glucocorticoids and disrupts neutrophil circadian rhythm and promotes neutrophil extracellular traps, which helps build a premetastatic environment [38]. Clinical data also show that patients with anxiety or depression have shorter PFS [39]. This study focuses exactly on key points of the psychoneuroimmune axis.

The unique advantage of the TSZA regimen is that it combines herbal treatment with psychological intervention. The main components of Compound Ciwujia Granules, such as eleutheroside, have been shown to regulate the HPA axis and increase brain-derived neurotrophic factor, so they improve cognitive function and emotional stability at the molecular level [40-42]. In addition, a single intervention is not enough to deal with complex cancer-related symptom clusters. For symptom clusters with emotional and cognitive problems, the effect of drug treatment is often limited. Given that standard psychological interventions such as CBT have strong evidence in oncology [43], they are integrated into our protocol to optimize therapeutic outcomes. This approach uses cognitive adjustment to rebuild coping strategies and improve psychological resilience, and it works together with Compound Ciwujia Granules to form a combined internal and external treatment model.

A key innovation of this study is the use of advanced methods to link TCM with modern science. In addition to treating the clinical symptoms of PNSC in ovarian cancer, this study also explores the biological mechanism of the TSZA regimen. We use fMRI to observe changes in functional connectivity in the limbic system, including the amygdala and hippocampus, to provide imaging evidence of symptom improvement and to show its effect on central neural circuits. We also test peripheral blood and tumor tissue to identify biomarkers related to PNSC relief at the molecular level. This approach combines macroscopic imaging and molecular data and supports the scientific basis of integrated TCM in managing cancer symptom clusters.

With the evolving understanding of the cancer symptom spectrum, symptom management is shifting from traditional supportive care to a key component influencing treatment outcomes [44]. PNSC not only impairs quality of life but also indirectly compromises antitumor efficacy by reducing treatment adherence and chemotherapy completion rates. In this context, this study can be regarded as an intervention targeting systemic anticancer therapy through a PNSC-based approach, rather than merely symptomatic management. We propose that alleviation of PNSC may be achieved by reducing systemic inflammatory burden, modulating the neuroendocrine-immune axis, and enhancing resilience to psychological stress. This approach may provide biological plausibility for the symptom-inflammation-immunity-tumor control continuum. Furthermore, the protocol follows the core idea of holistic oncology by integrating symptom management with antitumor therapy to optimize both survival and quality of life.

This study has several limitations. First, owing to technical difficulties in preparing a placebo for herbal medicine, a low-dose active comparator (containing 10% of the active components) is used instead. Although the control intervention is well matched to the investigational treatment in appearance, smell, and taste, it may retain partial pharmacological activity, potentially leading to an underestimation of the true treatment effect. Second, the generalizability of the findings from this study is somewhat limited. The study population is restricted to mainland Chinese patients with ovarian cancer who meet the TCM syndrome diagnosis of spleen-kidney yang deficiency. To ensure internal validity and robust causal inference, patients with a history of psychiatric disorders will be excluded. This measure aims to minimize confounding factors, ensuring that the observed PNSC is predominantly attributable to the malignancy and its treatment rather than a continuation of preexisting conditions. Furthermore, from an ethical and clinical safety perspective, patients with established psychiatric disorders require specialized care that exceeds the scope of this supportive intervention. While these criteria may limit the generalizability of the findings, they prioritize participant safety and the integrity of the study data. Third, the primary and most secondary end points are based on patient-reported outcomes; in the context of concomitant psychological interventions, response bias and performance effects cannot be fully excluded. Fourth, mechanistic exploratory analyses, including fMRI and multiomics, are conducted in a randomly selected subset, which may limit statistical power. Finally, although the 12-month follow-up is sufficient to assess symptom improvement, it may be inadequate to evaluate long-term OS.

In summary, the burden of psychological symptoms plays a critical role in tumor initiation, progression, and prognosis, underscoring the need for proactive psychoneurological symptom interventions in clinical practice. This study aims to provide high-quality evidence for the comprehensive management of PNSCs in patients with ovarian cancer. We anticipate that this study will not only improve the clinical manifestations of PNSCs and enhance patients’ quality of life but also strengthen antitumor immune responses and synergistically improve the efficacy of standard therapies, ultimately contributing to prolonged survival. In addition, the TSZA regimen, which integrates TCM with modern psychological interventions, may have broader applicability across other cancer types. This approach is expected to facilitate the integration of the biopsychosocial model into contemporary oncology and provide a practical framework for developing multidimensional intervention strategies.

## Abbreviations

AE: adverse event
CBT: cognitive behavioral therapy
ECG: electrocardiography
EDC: Electronic Data Capture
FAS: full analysis set
fMRI: functional magnetic resonance imaging
HPA: hypothalamic-pituitary-adrenal
MDASI-OC: MD Anderson Symptom Inventory for Ovarian Cancer
OS: overall survival
PFS: progression-free survival
PNSC: psychoneurological symptom cluster
SAE: serious adverse event
TCM: traditional Chinese medicine
TZSA: TiaoShenZhiAi
WHO: World Health Organization
